# Effects of early standardized management on the growth trajectory of offspring with gestational diabetes mellitus at 0–5 years old: a preliminary longitudinal study

**DOI:** 10.1038/s41598-023-40928-6

**Published:** 2023-08-25

**Authors:** Bingbing Guo, Jingjing Pei, Yin Xu, Yajie Wang, Xinye Jiang

**Affiliations:** Wuxi Maternity and Child Health Care Hospital, Women’s Hospital of Jiangnan University, Jiangnan University, Wuxi, 214002 Jiangsu China

**Keywords:** Emotion, Social neuroscience

## Abstract

To explore the application value of early standardized management in the delivery of neonates of pregnant women with gestational diabetes mellitus (GDM). Parturient diagnosed with GDM and their offspring were selected in our hospital from January 1, 2015 to December 31, 2017 to underwent early standardized management. Non-GDM pregnant women and their offspring were selected as the control group. The growth and development of children aged 0–5 years in the two groups were longitudinally followed up, and the mixed linear model was used to evaluate and compare the growth trajectories. There was no significant difference in height and weight between the two groups at 1 year old (*P* > 0.05), but the BMI of the GDM group was significantly higher than that in the control group. After 1 year of age, both groups of offspring were similar in height, weight, and BMI, and these similarities persisted at 2, 3, 4, and 5 years of age. After controlling for covariates, the weight, length/height of the two groups of children were slightly different in the growth trajectories between 0–1 years old, 1–2 years old, 2–3 years old, 3–4 years old, and 4–5 years old with no statistical significance (*P* > 0.05). Although growth differences between the two groups of children were detected within 1 year of age, there were no significant differences in growth trajectories from 1 to 5 years between two groups, which proved that early standardized management has positive significance.

## Introduction

Gestational diabetes mellitus (GDM) refers to the abnormal glucose metabolism of varying degrees first discovered or occurred during pregnancy, and seriously endangers the health of mothers and babies^1–3^. The incidence of GDM has continued to rise globally over the past few decades^4,5^. According to the International Diabetes Federation (IDF), approximately 21.3 million live births (16.2%) were affected by some form of hyperglycemia during pregnancy in 2017^6^. About 18.4 million of these cases were due to gestational diabetes mellitus (GDM), accounting for 86.4% of all hyperglycemia in pregnancy. The global incidence of GDM in 2017 was approximately 14%, with the highest primary prevalence of gestational hyperglycemia in Southeast Asia at 26.6% and the lowest in Africa at 9.5%. With economic development and improvement of people's living standards, multiple factors such as unhealthy lifestyles and excessive nutrition, and the improvement and standardization of prenatal screening techniques during pregnancy, the incidence of GDM in China is increasing year by year, which has attracted widespread attention. The prevalence of high-risk pregnant women, such as advanced age, pre-pregnancy overweight or obesity, has risen sharply and is likely to increase further with the implementation of policies to encourage fertility. The high incidence of GDM has brought a huge burden to China's healthcare system. According to the new International Association of Diabetes and Pregnancy Study Groups (IADPSG) criteria, the prevalence of GDM was as high as 19.7% among 15,194 pregnant women in 15 hospitals in Beijing, China in 2013^7^. A recent systematic review and meta-analysis including 25 cross-sectional or retrospective studies and 79,064 Chinese participants showed that the prevalence of GDM in mainland China was 14.8%^8^. Therefore, enough attention should be paid to GDM and its offspring, and early active intervention should be carried out.

As one of the pregnancy complications that seriously endangers maternal and infant health, a large number of studies have shown that GDM will not only increase the occurrence of maternal and infant perinatal complications (such as preeclampsia, premature birth, polyhydramnios, etc.), but also have long-term effect on obesity, abnormal glucose metabolism, hypertension and other diseases^9–12^. At present, offspring of GDM have not shown obvious signs of disease in the early stage, and they have not received enough attention and have entered the children's routine health care system. They have not received timely and effective attention and early intervention. However, interventions after observation of obesity, metabolic syndrome, and abnormal psycho-behavioral development are often ineffective. In addition, there are limited data on the long-term growth and development of GDM offspring with normative management. On the basis of previous research, we aimed to carry out early standardized management of pregnant women with GDM and their offspring, and longitudinally follow-up of the growth and development of GDM offspring aged 0–5 years old in early standardized management. We hypothesize that early standardized management can improve the growth trajectory of GDM offspring aged 0–5 years old.

## Materials and methods

### Subjects

Ninety neonates, whose mothers were diagnosed with GDM, born in our hospital from January 1, 2015 to December 31, 2017 were selected as the GDM group. A total of 90 neonates who were delivered by non-GDM pregnant women who received antenatal care in our hospital during the same period were selected as the control group. The basic information of the offspring since birth was collected, and their growth and development were followed up. This study was conducted according to the guidelines laid down in the Declaration of Helsinki and all procedures involving human subjects were approved by the Ethics Review Committee of Wuxi Maternity and Child Health Care Hospital (No: 2021-01-1215-32). Written informed consent was obtained from all subjects and their legal guardian.

GDM group: Inclusion criteria: Healthy before pregnancy, denied family history of hypertension and diabetes, developed gestational diabetes mellitus (GDM) in this pregnancy and regularly participated in pre-pregnancy examinations; The legal guardian of the child voluntarily accepted to enter the health management system. Exclusion criteria: Mothers with the following diseases in addition to GDM: twins and multiple births; thyroid dysfunction; liver and kidney diseases; tuberculosis, HIV and sexually transmitted diseases and other infectious diseases; moderate to severe anemia; chronic intestinal diseases; epilepsy; heart disease, etc.; clinical data is incomplete.

Control group: Inclusion criteria: Neonates born to mothers with normal glucose metabolism during pregnancy; with complete clinical data and informed consent of the guardian. Exclusion criteria: incomplete clinical data; Did not undergo oral glucose tolerance test during pregnancy.

Diagnostic criteria of GDM refer to the "Guidelines for the Diagnosis and Treatment of Diabetes in Pregnancy (2014)" formulated by the Obstetrics Group of the Obstetrics and Gynecology Branch of the Chinese Medical Association and the Gestational Diabetes Collaborative Group of the Perinatal Medicine Branch of the Chinese Medical Association^13,14^: The 75 g OGTT test was performed at the first visit at 24–28 weeks and after 28 weeks. Before the test, pregnant women were fasted for at least 8 h, and 75 g of glucose was orally administered within 5 min. The limits are: 5.1 mmol/L on an empty stomach, 10.0 mmol/L 1 h after taking sugar, and 8.5 mmol/L 2 h after taking sugar. GDM is diagnosed as any one of the three blood sugar levels that meets or exceeds the above criteria.

### Interventions

GDM group: Combined with relevant research consensus, a set of early standardized management and follow-up plans for GDM women and their offspring were formulated. For GDM women: Once diagnosed with GDM, regular follow-up guidance in specialized outpatient clinics were applied, and comprehensive guidance and regular follow-up will be given in terms of diet, exercise, blood sugar monitoring, and insulin treatment during pregnancy. Within 6 months after giving birth, education on exclusive breastfeeding was conducted and breastfeeding was encouraged to 2 years of age. After 6 months of childbirth, mothers were given instruction on the reasonable addition of complementary foods to the test children, and a “Baby Kitchen” complementary food addition course was offered every 1 to 2 months to demonstrate the preparation of complementary foods and guide reasonable food transitions. At the same time, parent schools were held regularly to teach infants and young children the laws of feeding, exercise, and intellectual development, and to guide and train them in terms of exercise, feeding, language, cognition, life, and communication skills.

For offspring, we developed a systematic early comprehensive management program: Follow-up once a month within 1 month after birth, including physical development assessment, newborn hearing screening, developmental screening, glaucoma screening, etc. Follow-up once a month from 1 to 6 months to assess physical growth and development, and provide feeding consultation. Follow-up once every 2 months from 6 to 12 months to conduct motor development assessment and provide parent–child exercise guidance. Follow-up once every 3 months after 1 year of age to assess whether growth and development are normal, and early intervention will be performed in case of abnormalities. Early matching interventions were performed on high-risk children based on screening results.

Control group: Participate in the "Children's Health Management Service for 0–6 Years Old" in accordance with the requirements of the national basic public health service standards.

### Anthropometric measurements

All subjects included in this study were measured within 24–48 h after birth by trained and qualified researchers using uniform equipment, including height, weight, head circumference, upper arm circumference, biceps, triceps, Skinfold thickness of the subscapular, anterior and upper four parts of the iliac. The weight and length of infants and young children under 2 years of age were measured by infant length–weight scale. The weight reading was accurate to 10 g, and the length reading was accurate to 0.1 cm. Children over 2 years old were measured with a height scale (maximum weighing 120 kg, minimum division 0.5 kg; height measurement range 70–190 cm, division value 0.5 cm). The head circumference, chest circumference, and upper arm circumference were measured with a soft ruler, and the readings were accurate to 0.1 cm. The skinfold thickness was measured using a skinfold thickness gauge, which was calibrated before each measurement, accurate to 0.5 mm, and the pressure was 10 g/mm^2^. Body fat percentage, body fat content and lean body mass were calculated according to the Weststrate method^15^. Each index was measured 3 times and the average value was taken.

### Baseline information collection

Using a self-made questionnaire, we collected data on the baby's birth status, mother's prenatal conditions, and basic demographic data of the family. This includes baby's gender, birth weight, birth length, mother's weight gain during pregnancy, complications during pregnancy, nutrient intake during pregnancy, parents' age, parents' height, parents' weight, level of education, total monthly family income, type of family, and family economic status, among other statistical data.

### Statistical analysis

Data was collected by EpiData for double entry and logical check, and SAS 9.4 (SAS Institute Inc, Cary, NC) was used for statistical analysis. Statistical description of measurement data was carried out by x ± SD, and t-test was used to compare the mean between groups when it obeyed normal distribution or approximately normal distribution and the variance was homogeneous, and t' test was applied when the variance was unequal. Data were statistically described, and statistical analysis was performed using the χ2 test, Fisher's exact test, or the continuity-adjusted χ2 test. The influencing factors of body fat percentage of newborns were analyzed by multiple linear stepwise regression analysis^16,17^. *P* < 0.05 was used as the criterion to judge whether the difference was statistically significant. Visual inspection of the histogram and Shapiro–Wilk W test for normality of variable distributions-test. The primary analysis used mixed linear models for offspring height (unnormalized and normalized) and body weight growth trajectories. Different baseline levels between the two groups were included as covariates, including maternal pre-pregnancy BMI, pregnancy weight gain, mode of delivery, and mother's age at delivery. The primary concern was whether the change in outcome over 5 years was significantly different between the two groups, which was assessed by linear comparisons.

### Ethics approval and consent to participate

This study was approved by the Ethics Review Committee of Wuxi Maternity and Child Health Care Hospital (No: 2023-06-0804-34), and informed consent was obtained from all subjects and their legal guardian. All methods were carried out in accordance with relevant guidelines and regulations.

## Results

### Basic information of the mothers

There were no significant differences in maternal age, height, prenatal weight, pregnancy number, parity, mode of delivery, education level and monthly household income between the GDM group and the control group (*P* > 0.05). The proportions of weight gain during pregnancy and family history of diabetes were higher than those in the control group, and the differences were statistically significant (*P* < 0.05), see Table 1.

**Table 1:** Comparison of the basic information of two groups [x ± s or number of cases (%)].

	GDM	Control	*t*/*χ*^*2*^	*P*
Age	29.29 ± 3.78	28.53 ± 3.71	1.35	0.18
Height (cm)	160.8 ± 4.73	161.7 ± 4.60	1.29	0.20
Pre-pregnancy weight (kg)	56.22 ± 8.56	53.14 ± 6.94	2.65	< 0.01
Prenatal weight (kg)	71.01 ± 8.85	69.35 ± 7.86	1.33	0.18
Pre-pregnancy BMI (kg/m^2^)	21.72 ± 2.92	20.31 ± 2.30	3.59	< 0.01
Weight gain (kg)	16.21 ± 3.98	14.89 ± 4.27	2.31	0.02
Pregnancy			0.20	0.65
1	44 (48.9)	47 (52.2)		
> 1	46 (51.1)	43 (47.8)		
Parity*			1.41	0.23
1	27 (30.0)	20 (22.2)		
> 1	63 (70.0)	70 (77.8)		
Mode of delivery			0.02	0.88
Natural delivery	40 (44.4)	41 (45.6)		
Cesarean section	50 (55.6)	49 (54.4)		
History of diabetes*			5.76	0.02
Yes	15 (16.7)	4 (4.5)		
No	75 (83.3)	85 (95.5)		
Education level			0.10	0.95
Junior high school and below	8 (9.0)	7 (7.8)		
High and secondary school	10 (11.2)	10 (11.1)		
College and above	71 (79.8)	73 (81.1)		
Monthly household income (Yuan)			1.11	0.57
< 5000	23 (26.7)	28 (32.9)		
5000–10,000	40 (46.5)	39 (45.9)		
> 10,000	23 (26.8)	18 (21.2)		

Note: Except for * which used the χ2 test, all other comparisons were conducted using t-tests. ^#^t 'test was used to compare the mean of two samples with uneven variance.

### Anthropometric indexes of offspring at birth

Birth weight (3.4 ± 0.5 vs. 3.3 ± 0.4 kg; *P* = 0.03), chest circumference (33.4 ± 1.5 vs. 32.9 ± 1.3; *P* = 0.03), and BMI (12.5 ± 1.5 vs. 12.8 ± 1.5 vs. 12.9 ± 1.2 kg /cm^2^; *P* = 0.02) of GDM offspring were higher than that in control group. There was no significant difference in birth gestational age, birth length, head circumference and upper arm circumference between the GDM group and the control group (*P* > 0.05). The skinfold thicknesses of the biceps brachii, triceps brachii, subscapular angle and upper iliac crest in the GDM group were significantly higher than those in the control group (*P* < 0.01). The weight in the GDM group was 0.1 kg (*P* = 0.03) and 3.1% higher BF% (*P* < 0.01), and the delipidated mass in the GDM group was 0.1 kg lower than that in the control group (*P* = 0.03), see Table 2.

**Table 2:** Comparison of anthropometric measures at birth between the two groups.

	GDM	Control	*t/ t'*	*P*
Birth status				
Gestational age (weeks)	38.5 ± 1.6	38.8 ± 1.3	1.21	0.22
Weight (kg)	3.4 ± 0.5	3.3 ± 0.4	2.16	0.03
Length (cm)	50.0 ± 0.6	49.8 ± 0.7	1.55	0.12
BMI (kg/cm^2^)	13.7 ± 1.6	13.2 ± 1.3	2.08	0.04
Head circumference (cm)	34.1 ± 1.2	34.2 ± 1.2	0.51	0.61
Chest circumference (cm)	33.4 ± 1.5	32.9 ± 1.3	2.17	0.03
Upper arm circumference	11.2 ± 1.3	11.3 ± 0.8	0.68	0.67
Skinfold thickness				
Biceps (cm)	4.2 ± 1.1	3.6 ± 0.8	4.65^#^	< 0.01
Triceps (cm)	6.2 ± 1.5	5.1 ± 1.0	5.46^#^	< 0.01
Subscapular angle (cm)	5.6 ± 1.4	4.8 ± 0.9	4.45^#^	< 0.01
Upper iliac crest (cm)	4.3 ± 1.2	3.4 ± 0.8	5.58^#^	< 0.01
Body fat percentage (BF%)	17.9 ± 3.4	14.8 ± 3.2	6.26	< 0.01
Body fat content (kg)	0.6 ± 0.2	0.5 ± 0.2	4.22	< 0.01
Delipidated mass (kg)	2.8 ± 0.3	2.9 ± 0.3	2.16	0.03

Note: ^#^t' test was uesdto compare the mean of two samples with uneven variance.

### Comparison of growth trajectories between two groups of children aged 0–5 years

**Figure 1:**
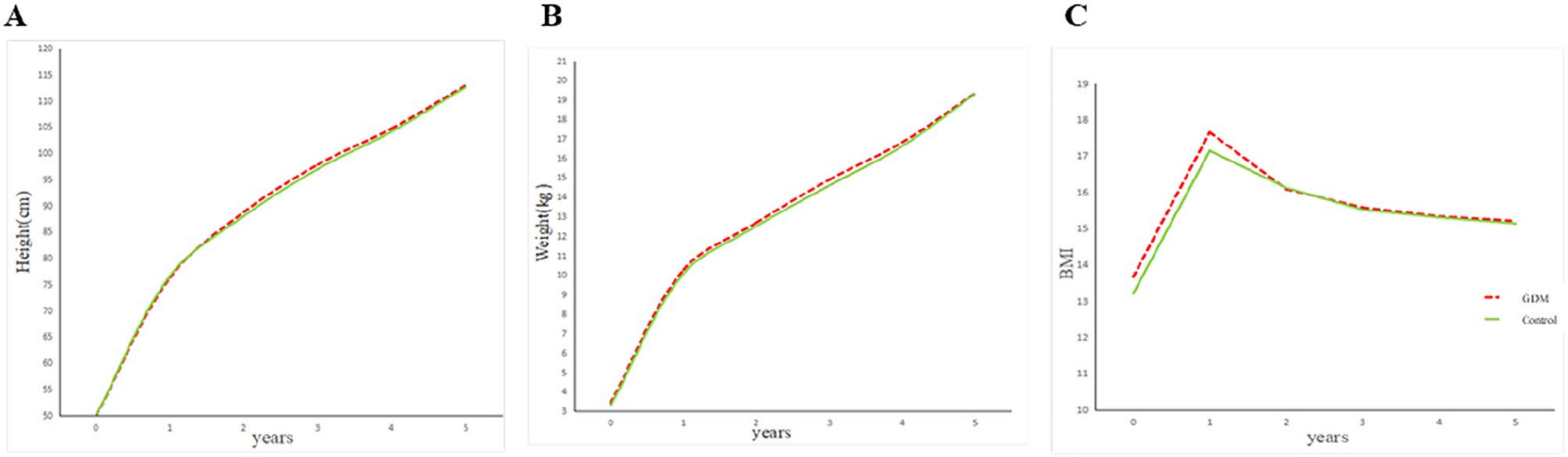
The growth curve of height and weight aged 0–5 years. A: Growth curves for the height of two groups of children aged 0–5; B: Growth curves for the weight of two groups of children aged 0–5; C: Growth curves for the BMI (Body Mass Index) of two groups of children aged 0–5.

Although there was an initial difference at birth and persisted to about 1 year old, there was no significant difference in height and weight between the two groups at 1 year old, and the BMI of the children in the GDM group was significantly higher than that of the children in the control group (17.65 ± 1.43 vs. 17.14 ± 1.22 kg/cm^2^, *P* = 0.01). But after 1 year of age, GDM offspring and control offspring were similar in height, weight, and BMI, which persisted at 2, 3, 4, and 5 years of age (Table 3). From Fig. 1A and B, we can see that the growth trajectories of height and weight for the two groups of children are basically consistent. Figure 1C shows that the BMI of children in the GDM group is higher than that of the control group from birth to 1 year old. From ages 2 to 5, the BMI curves of the two groups tend to converge.

**Table 3:** Comparison of anthropometric indexes of offspring between GDM group and control group (x ± s).

	Age	GDM	Control	*P*
Height (cm)	1	76.64 ± 2.52	76.28 ± 2.43	0.33
	2	88.74 ± 2.81	87.99 ± 3.16	0.09
	3	97.79 ± 3.13	96.97 ± 3.65	0.11
	4	104.7 ± 3.73	104.1 ± 4.27	0.34
	5	112.9 ± 4.52	112.6 ± 4.47	0.65
Weight (kg)	1	10.28 ± 1.14	10.08 ± 0.99	0.19
	2	12.68 ± 1.13	12.50 ± 1.34	0.37
	3	14.89 ± 1.29	14.59 ± 1.51	0.15
	4	16.81 ± 1.64	16.61 ± 1.91	0.45
	5	19.34 ± 3.15	19.31 ± 2.18	0.95
BMI (kg/cm^2^)	1	17.65 ± 1.43	17.14 ± 1.22	0.01
	2	16.08 ± 1.19	16.12 ± 1.22	0.82
	3	15.57 ± 1.05	15.51 ± 1.25	0.72
	4	15.33 ± 1.02	15.30 ± 1.15	0.86
	5	15.20 ± 1.77	15.13 ± 1.18	0.78

We next assessed differences in height and weight gain between the two groups after adjusting for different baseline covariates (mother's pre-pregnancy BMI, gestational weight gain, mode of delivery, mother's age at birth, birth weight, and birth length). The results showed that the weight, length/height growth trajectories of the two groups of offspring were slightly different between 0–1 years old, 1–2 years old, 2–3 years old, 3–4 years old, and 4–5 years old with no statistical significance (*P* > 0.05). See Table 4.

**Table 4:** Growth trajectories of two groups of children aged 0–5 years.

	GDM (n = 90)	Control (n = 90)	*P*	GDM versus Control
	Means (95% CI)			Means (95% CI)
Weight gain (kg/month)				
0–1 years old	0.57 (0.55, 0.59)	0.57 (0.55, 0.59)	0.77	− 0.00 (− 0.03, 0.02)
1–2 years old	0.21 (0.19, 0.22)	0.19 (0.18, 0.21)	0.11	0.02 (− 0.00, 0.04)
2–3 years old	0.19 (0.17, 0.20)	0.17 (0.16, 0.19)	0.15	0.01 (− 0.01, 0.03)
3–4 years old	0.16 (0.15, 0.19)	0.15 (0.14, 0.18)	0.24	0.02 (− 0.01, 0.04)
4–5 years old	0.20 (0.17, 0.22)	0.18 (0.16, 0.21)	0.40	0.01 (− 0.02, 0.05)
Length/heigh increase (cm/month)				
0–1 years old	2.19 (2.15, 2.24)	2.24 (2.19, 2.28)	0.17	− 0.04 (− 0.11–0.02)
1–2 years old	1.00 (0.96, 1.04)	0.97 (0.94, 1.02)	0.34	0.03 (− 0.02, 0.08)
2–3 years old	0.74 (0.70, 0.77)	0.75 (0.72, 0.79)	0.53	− 0.01 (− 0.06, 0.03)
3–4 years old	0.62 (0.58, 0.67)	0.57 (0.52, 0.61)	0.06	0.06 (− 0.02, 0.12)
4–5 years old	0.63 (0.59, 0.66)	0.60 (0.57, 0.64)	0.29	0.03 (− 0.02, 0.08)

## Discussion

With the in-depth study of the theory of Development of Health and Disease (DOHaD), the influence of GDM on the long-term and offspring of pregnant women has attracted more and more attention. GDM not only has adverse effects on the mother and child during the perinatal period, the abnormal intrauterine environment of GDM may also increase the risk of obesity, hypertension, abnormal glucose metabolism, neurological diseases and cardiovascular diseases in the offspring in the long-term^18,19^. The Hyperglycemia and Adverse Pregnancy Outcomes (HAPO) Follow-up Study Group reported that GDM determined by the IADPSGcriteria after adjustment for maternal BMI was associated with increased body fat content and obesity in offspring aged 10–14 years^20^. Maternal blood glucose levels were inversely associated with insulin sensitivity and β-cell function, which results in a higher prevalence of impaired glucose tolerance in offspring of GDM mothers^16,17^. Zhao et al.^21^ conducted a cross-sectional study of 4740 9–11-year-old children in 12 countries and showed that the risk of obesity in the offspring of GDM was 1.53 times higher than that of non-GDM. A population-based cohort study found that in utero exposure to gestational diabetes was significantly associated with the development of autism spectrum disorder in offspring^22^. Regardless of glycemic control during pregnancy, the effects of GDM on offspring are not limited to the intrauterine and neonatal periods, and may persist into children, adolescents, and even adults. Therefore, early systematic management of offspring with GDM is of great significance and can affect health status and quality of life throughout life, which is more active, proactive and effective than intervening in adults.

Some studies have pointed out that children are not included in the standardized health management system is one of the main reasons for the deviation of the growth and development of GDM offspring^23^. The International Federation of Gynecology and Obstetrics (FIGO) proposed in the GDM guidelines issued in 2015 that the follow-up of GDM women and their offspring should be combined with routine physical examination and vaccination of offspring^24^. Our results showed that the birth weight (3.4 ± 0.5 vs. 3.3 ± 0.4 kg; *P* = 0.03), BMI (12.5 ± 1.5 vs. 12.8 ± 1.5 vs. 12.9 ± 1.2 kg/cm^2^; *P* = 0.02), body fat percentage (BF%; 17.9% vs. 14.8%, *P < *0.01), and body fat mass (0.6 kg vs. 0.5 kg; *P < *0.01) of GDM offspring were significantly higher than those in the control group, which was consistent with other studies^25–27^. Hyperglycemia that occurs in the second and third trimesters increases maternal blood concentrations of amino acids and fatty acids and transports excess nutrients to the fetus through the placenta^28^. Excessive nutrient supply stimulates fetal pancreatic β-cells to increase insulin secretion. Fetal hyperinsulinemia can lead to overgrowth of insulin-sensitive tissues such as the liver, adipose tissue, and heart.

Despite almost no evidence suggesting that routine treatment of GDM affects the long-term outcomes of offspring. In a follow-up study of children (ages 5–10) born to women who participated in a multicenter trial, there was no reduction found in obesity or metabolic dysfunction in the offspring of women who received treatment (diet therapy and insulin if necessary) compared to those with mild untreated GDM^29^. However, lower fasting blood glucose levels were observed only in female offspring. In this study, although growth differences were detected at birth and persisted up to 1 year of age, we observed similar growth and development trends between 1 and 5 years between offspring of GDM and control group after early standardized management, which suggests that early management should be initiated once a pregnant woman with GDM is identified, and early integrated management of the offspring should be initiated. This is consistent with the results of other studies. Zhou et al. found that the number of blood glucose abnormalities in pregnant women with GDM is directly proportional to the occurrence of neonatal neurological abnormalities, and strengthen prenatal healthcare management for pregnant women could reduce the incidence of abnormal neonatal neurological development^30^. Although the majority of obesity in adolescents and adults occurs in individuals with normal birth weight, the outcomes of early childhood growth remain clinically relevant because a large proportion of adolescent obesity is established before the age of 5 years. Children who are overweight in kindergarten are four times more likely to become obese by eighth grade than children of normal weight^31^.

Although this study indicated the importance of early standardized management of GDM offspring, there were still some shortcomings. Selection bias may have arisen because some data were not collected completely or some infants could not be included in this study. In addition, the number of follow-up cases was relatively small, and it was necessary to further expand the sample size and increase the research content, such as children's body composition, in order to better evaluate the physical development level.

## Data Availability

The dataset generated during and analysed during the current study is available from the corresponding author on reasonable request.
